# Assessment of Visual Attention in Teams with or without Dedicated Team Leaders: A Neonatal Simulation-Based Pilot Randomised Cross-Over Trial Utilising Low-Cost Eye-Tracking Technology

**DOI:** 10.3390/children11081023

**Published:** 2024-08-21

**Authors:** Prakash Kannan Loganathan, Anip Garg, Robert McNicol, Conor Wall, Matthew Pointon, Peter McMeekin, Alan Godfrey, Michael Wagner, Charles Christoph Roehr

**Affiliations:** 1 Neonatal Intensive Care Unit, The James Cook University Hospital, Middlesbrough TS4 3BW, UK; anip.garg@nhs.net; 2 Clinical Academic Office, Faculty of Medical Sciences, Newcastle University, Newcastle upon Tyne NE1 7RU, UK; 3 Department of Physics, University of Durham, Durham DH1 3LE, UK; 4 Department of Computer and Information Sciences, Northumbria University, Newcastle upon Tyne NE1 8ST, UK; robertmcnicol@gmail.com (R.M.); conor3.wall@northumbria.ac.uk (C.W.); m.pointon@northumbria.ac.uk (M.P.); alan.godfrey@northumbria.ac.uk (A.G.); 5 Department of Nursing, Midwifery, and Health, Northumbria University, Newcastle upon Tyne NE1 8ST, UK; peter.mcmeekin@northumbria.ac.uk; 6 Division of Neonatology, Pediatric Intensive Care and Neuropediatrics, Department of Pediatrics, Comprehensive Center for Pediatrics, Medical University of Vienna, 1090 Vienna, Austria; michael.b.wagner@meduniwien.ac.at; 7 National Perinatal Epidemiology Unit, Medical Sciences Division, Nuffield Department of Population Health, University of Oxford, Oxford OX1 2JD, UK; charles.roehr@npeu.ox.ac.uk; 8 Newborn Services, Southmead Hospital, North Bristol Trust, Bristol BS10 5NB, UK; 9 Faculty of Health Sciences, University of Bristol, Bristol BS8 1QU, UK

**Keywords:** human factors, neonatal resuscitation, visual attention, eye tracking, team performance

## Abstract

Background: Eye-tracking technology could be used to study human factors during teamwork. Objectives: This work aimed to compare the visual attention (VA) of a team member acting as both a team leader and managing the airway, compared to a team member performing the focused task of managing the airway in the presence of a dedicated team leader. This work also aimed to report differences in team performance, behavioural skills, and workload between the two groups using validated tools. Methods: We conducted a simulation-based, pilot randomised controlled study. The participants included were volunteer paediatric trainees, nurse practitioners, and neonatal nurses. Three teams consisting of four team members were formed. Each team participated in two identical neonatal resuscitation simulation scenarios in a random order, once with and once without a team leader. Using a commercially available eye-tracking device, we analysed VA regarding attention to (1) a manikin, (2) a colleague, and (3) a monitor. Only the trainee who was the airway operator would wear eye-tracking glasses in both simulations. Results: In total, 6 simulation scenarios and 24 individual role allocations were analysed. Participants in a no-team-leader capacity had a greater number of total fixations on manikin and monitors, though this was not significant. There were no significant differences in team performance, behavioural skills, and individual workload. Physical demand was reported as significantly higher by participants in the group without a team leader. During debriefing, all the teams expressed their preference for having a dedicated team leader. Conclusion: In our pilot study using low-cost technology, we could not demonstrate the difference in VA with the presence of a team leader.

## 1. Background

Neonatal resuscitation training has been shown to decrease neonatal mortalities and morbidities but there is substantial scope for further improvement [1,2]. Despite designated neonatal resuscitation training programmes [1,3], neonatal resuscitations teams continue to make errors in 23% to 44% of neonatal resuscitations [4]. Many resuscitation council guidelines have emphasised the importance of studying human factors during neonatal resuscitation [1,5]. Human factors in health care involve enhancing clinical performance through an understanding of the effects of teamwork, tasks, equipment, workspace, culture, and organisation on human behaviour in clinical settings [6]. Despite the recommendations, there is a dearth of studies examining human factors [7].

Ideally, teams attending neonatal resuscitations should be led by a senior leader who could primarily focus on team management and decision-making. Having a dedicated resuscitation leader who has a single dedicated role of managing the team/situation could improve overall situational awareness and resuscitation outcomes. In practice, owing to strains on personnel availability, there could be situations where the designated leader is often forced to take on additional clinical tasks such as intubation. In dynamic, time-critical situations such as neonatal resuscitation, multi-tasking may lead to human error, a loss/reduction in situational awareness, ineffective communication, and sub-optimal performance [8].

Eye-tracking technology could provide a standardised objective way of assessing human behaviour in resuscitation simulation scenarios [9,10,11,12,13,14]. Eye-tracking is a method of observing eye movement as a reflection of attentional behaviour [15]. In recent years, it has been used in the medical field and is a valuable tool in medical education by providing training and effective feedback [15,16,17].

The primary objective of our pilot study was to compare the visual attention (VA) of a team member when the person is managing the airway and acting as a team leader (both roles played by the same person) as compared to having a dedicated team leader and the same team member only having to perform airway management. VA was defined by eye-tracking metrics providing fixation and dwell time data on areas of interest (AOIs). We used VA as our primary outcome based on previously published literature [9,11]. A fixation is a point where the visual gaze remains at a particular location. This includes fixations and revisits conveying the level of interest. Dwell time is a measure of the length of time it takes an individual to comprehend the information being fixated on [18]; the greater the dwell time, the greater the level of interest. The area that the operator is interested in gaining information about defines the AOI. The secondary objectives were the difference in team performance, behavioural skills, and workload using a standardised tool. This was a pilot study to understand the feasibility of using low-cost eye-tracking technology for studying human behaviours in neonatal simulation.

## 2. Methods

### 2.1. Settings

We conducted a single-centre, simulation-based, pilot cross-over randomised controlled study in a single centre. The entire simulation study was conducted in a dedicated immersive simulation suite providing a standard delivery room/operating theatre environment.

### 2.2. Participants

We included volunteer paediatric speciality trainees (any level) or advanced neonatal nurse practitioners (ANNPs) who have worked at least 6 months in NICUs and nursing staff (any level) who were currently working in the NICU. To be included, all of the participants needed a valid Neonatal Life Support (NLS) certification. All potential participants were contacted by email and by displaying information leaflets in our NICU. Based on the participants’ availability and their preferred timing on the day of simulation, we formed three teams, each of the teams consisting of a total of four members with one neonatal nurse, one junior trainee working in the junior rota, and two senior trainees working in the senior rota. We collected personal data on participant professional training and neonatal experience before the simulations.

### 2.3. Simulation

Before each simulation, participants received a briefing about the features of the simulation manikins. We used low-fidelity Nena Sim-Term and preterm manikins which allow intubation as well as mask ventilation (Medical-X, Rotterdam, The Netherlands). Before each scenario, teams were given time to prepare for the simulation: role allocation, equipment checking, and escalation plan. Each of the teams participated in two simulation scenarios: one with the management of an extreme preterm infant and another with the management of a term newborn. Details of the term and preterm resuscitation scenarios are provided in Supplementary File S2. Both simulation scenarios included the need for endotracheal intubation. In the term baby scenario, the provision of chest compression and umbilical vein administration of epinephrine was required. The two scenarios were the same for all participants. We ran the two simulation scenarios with three different teams, giving a total of six scenarios with each scenario running approximately for 10–15 min. After completing each scenario, all members of the team were provided with an immediate debrief by the same person who had experience as an NLS instructor for more than 20 years (E.S.). The person who provided debriefs was not involved in the outcome assessment.

### 2.4. Study Algorithm

Each team underwent one scenario with a team leader and another scenario without a team leader. Whether the first simulation scenario should be a “preterm or term scenario” and whether the team should have a team leader or not was decided randomly by tossing a coin. Similarly, the selection of who would be the team leader among the two senior trainees was decided by tossing a coin. The other senior trainee would remain as the airway operator in both simulations. Only the trainee who was the airway operator would wear eye-tracking glasses and not any other participants. Following this, the sequence in which each team was allocated with/without a team leader was alternated with each team (cross-over). For example, if Team 1 started with the team leader for their “preterm scenario,” (all the four members of the team would participate), subsequently the same team would participate in the “term scenario” without the team leader (only three members of the team would participate) with same person managing the airway (Supplementary Figure S1). This ensured that both groups had an equal number of scenarios with (n = 3) and without a team leader (n = 3). All the airway operators were briefed about using eye-tracking wearables and underwent a pre-simulation test for familiarity and calibration (Supplementary Figure S2). The study algorithm and sequence of simulation and team allocation are provided in Supplementary File S2. The study protocol is provided as Supplementary File S1.

### 2.5. Visual Attention (Situation Awareness)

For eye-tracking measurements, we used the “Pupil Labs, Core Eye Tracker,” Berlin, Germany: 160 × 51 mm, high speed 120 Hz and 200 Hz, (https://pupil-labs.com/products/core accessed on 13 November 2023). This eye tracker is low-cost (approximately GBP 3K), provides effective eye-tracking functionality, and comes with the Pupil Player software. All the data were stored on a secure server where further analysis of those data was conducted using a custom-made MATLAB^®^ (software version: R2024a; MathWorks Inc, Natick, MA, USA) algorithm [19,20]. To determine the total number of fixations and dwell times between the areas of interest (AOIs) for each of the videos, i.e., the baby, colleague, and monitor, the use of the Pupil Players post-hoc calibration functionality was deployed. We chose the AOI based on the study by Wagner et al. [11].

### 2.6. Team Performance and Behavioural Skills

In real-time, two investigators (V.N. and C.B.), who are both NLS instructors, independently assessed each scenario for team performance. We used a validated modified neonatal resuscitation performance evaluation (NRPE) tool for assessment [21]. This tool was originally developed based on the recommendations of the Neonatal Resuscitation Programme, American Heart Association. NRPE provides a categorical score (yes = 1 and no/incomplete action = 0) for each of the actions. It marks the performance under three domains: (i) appropriateness of decision-making, (ii) technical skills, and (iii) time taken to perform the appropriate resuscitative measures. It has seven subdomains: (i) preparation and initial steps, (ii) communication of heart rate, (iii) bag/mask ventilation, (iv) chest compressions, (v) intubation, (vi) medication administration, and (vii) umbilical vessel catheterisation. The results from this tool are valid and have a high inter-rater reliability [22].

We have modified the NRPE tool to match recommendations for neonatal life support in the UK (Supplementary File S2). We calculated the inter-rater reliability between the two independent NLS instructors.

The team’s behavioural performance evaluation was assessed using the behaviour assessment tool (BAT) by the same two NLS instructors (VN and CB). This tool has a 5-point rating for assessing 10 key behavioural skills: knowledge of the environment, anticipation and planning, assumption of a leadership role, communication among team members, distribution of workload, attention allocation, utilisation of information, utilisation of resources, calling for help, and professional behaviour (Supplementary File S2). The validity and reliability of this tool have been established previously [23].

### 2.7. Workload

For the assessment of workload, we used the National Aeronautics and Space Administration Task Load Index (NASA-TLX) [24]. This scoring system includes 6 scales on how an individual experiences work demands in various dimensions: mental demand (MD), physical demand (PD), temporal demand (TD), frustration (FR), effort (EF), and performance (PE) (Supplementary File S2). The reliability and validity of NASA-TLX have been demonstrated in studies [25,26]. We used raw scores of NASA-TLX rather than weighted scores as the raw score is more time-efficient and simpler to apply [27]. After completing each simulation, participants were asked to complete NASA-TLX forms. For each of the six domains, the participants marked their feelings on a scale of 1 to 20 in each domain, with 20 being the worst experience and 1 being the best experience.

### 2.8. Sample Size and Statistics

We had a convenience sample of three teams (with 6 six simulation scenarios) fit in to one day of study with volunteers. We used descriptive statistics for population characteristics. Categorical variables were presented as proportions, while numerical variables were presented as the mean with the standard deviation (SD) or the median with the interquartile range (IQR) as appropriate. For paired continuous outcomes, a paired *t*-test or Wilcoxon Signed Rank Test was used based on the data distribution. A *p*-value of <0.05 was considered statistically significant. Data were analysed in SPSS (v23, IBM) and R studio (R. RStudio, Boston, MA, USA).

Ethics committee exemption was provided, as the study took place in a simulation setting. All the staff who participated in the study were provided with a participant information sheet (PIS) concerning the study and completed an informed consent form and a short survey (Supplementary File S2).

## 3. Results

There were a total of 3 teams with 4 members in each team, totalling 12 participants. Table 1 provides the participants’ professional training and neonatal experience. Most of the staff had good prior neonatal resuscitation real-life experiences with a relatively smaller number of simulation experiences. All three of the participants who were airway operators and wearing eye-tracking glasses in the no-team-leader group had a greater number of total fixations on the AOIs manikin and on the monitors, though this was not statistically significant (Table 2). Participants in the no-team-leader group had a longer dwell time on colleagues than participants in the team leader group, though this was not statistically significant. Data on the number of fixations and fixation time for each participant and scenario are provided as Supplementary data (Excel file). There were no significant differences between the two groups regarding team performance: NRPE decision, NRPE techniques, NRPE total scores, and BAT scores (Table 3). Overall, there was no significant differences between the two groups with regards to primary and secondary outcomes. Scores for each individual scenario and by each assessor are provided in Supplementary Table S1.

For an arbitrarily chosen NRPE score of 80 and BAT score of 80%, the Kappa’s agreement between the two assessors was 100% and 33%, respectively. Table 4 provides the self-reporting on the workload. Except for physical demand which was reported to be significantly higher overall for participants in the group without a team leader, there was no difference in any of the domains among the participants. Participants who were airway operators and wearing eye-tracking glasses reported lower scores in all domains of workload with the presence of a team leader (Supplementary Table S2). The individual NASA Task Load Index scores (after excluding airway operators) are provided in Supplementary Table S3.

All three members wearing eye-tracking glasses reported that wearing eye-tracking glasses were not distracting and they would be able wear them in real-life situations. A few important points were discussed during our debrief sessions. For all the simulations, teams felt it was “easier to have a separately allocated team leader and not managing so many things.” In teams without a dedicated team leader, the senior person placed themselves in control of the airway and acted as team leader. One participant commented that it can be challenging when you have two members of the team with the same level of experience. The debriefer observed that the directions/instructions of the team were affected by confidence and the experience of the team leader. Input from the nursing staff varied depending on the confidence of the team leader. The debriefer also observed that familiarity among the staff could have some impact on trust between team members and their performance.

## 4. Discussion

In this pilot study, we compared aspects of human factors associated with neonatal resuscitation in a simulation setup. Visual attention as the primary outcome could also be an effective tool to measure individual performance (skills) in medical sciences [28]. It has been shown that an individual’s attention and thoughts corresponds to their point of fixation at any given time point [18]. Also, we focused on the team’s performance, behavioural skills, and task load using validated tools. Low-cost technology could potentially help in the widespread use in real-life resuscitation scenarios even in resource-limited settings.

Our study did not show any significant difference between the groups, though our sample size does not have adequate power to evaluate the teams’ performance, behavioural skills, and task load. We hypothesised that the experiences and personal attributes of the individual team members, team composition, and familiarity could be the possible reasons for our results. It was not possible to analyse these factors with our pilot study. For example, with one of our teams, both the airway person and the team leader were of the same level of experience and had an experienced neonatal nurse practitioner in their junior role. It is possible that, on occasion, leadership actions such as decision-making could have been taken by the experienced team member rather than supposed “team leader.”

Compared to paediatric trainees, who usually rotate to different hospitals every six months in the United Kingdom, neonatal nurse practitioners and neonatal nurses work in the same neonatal intensive care environment. Teams with neonatal nurse practitioners are highly likely to have better inter-professional familiarity. In a recently published RCT, a significant increase in team performance, communication, and psychological safety by increasing inter-professional familiarity was reported [29].

Dwell times among the simulation scenarios were variable and this could be due to the fact that the term scenario needed multiple interventions to be performed. The length of the simulation scenarios could have potentially influenced the VA. Our study showed higher physical demand in the group without a team leader; this could be due to the absence of one extra team member in the group. Our study had relatively high NRPE scores compared to other reported studies [21,30]. This could be due to a few possible reasons: we used two simulation scenarios that are commonly used in NLS courses, and the team was prepared well in advance for the simulation experiment. Also, both experienced assessors independently attained high NRPE and BAT scores.

Wearable eye-tracking glasses have two cameras, one capturing the reflected infrared light tracking pupillary movement and another camera capturing video from the participant’s viewpoint [9]. To date, wearable eye-tracking glasses have been used in a few neonatal and critical care studies [9,11,31,32]. A study to examine human factors during neonatal intubation in the neonatal unit found that 50% of visual attention is directed away from the infant and the team when they used nonstandard communication [10]. Previous studies have shown that novice trainees focus more on the monitoring system rather than the patient/baby [11].

Our study had several strengths. Response process validity controlling for potential sources of error with the administration was minimised by using a standardised simulation environment, using the same simulation scenarios, maintaining a similar team structure for all the simulations, and assessment by the same experienced investigators for assessing all the outcomes. Our study also had a few limitations including a small sample size. We could not control for individual team members’ experience and individual attributes, which could have affected the results. Due to practical issues, we recruited staff volunteers available on that study day which would have caused recruitment bias. Debriefing after each simulation scenario could have influenced the team’s performance in the subsequent scenario. Another limitation of eye-tracking is that it does not capture the situations of “inattentional blindness.” Meaning the operator may be looking at some aspect without putting their mind to it. In our study, we were not able to show specific differences at different time points of interest (i.e., chest compression) but rather only calculated an overall VA which could have impacted the results. We assessed only the VA of the airway operator and did not assess the performance/VA of the team leader who has other responsibilities like the delegation of roles, clinical decisions, and team management throughout the resuscitation process.

Our study results should not be interpreted that having a dedicated team leader does not improve team performance, as our study was not powered highly enough to answer all of these questions. Moreover, there was a self-reporting of increased physical demand in the group without a team leader, and with debriefing all the team felt the need for a separately allocated team leader. These results could be explored in future studies with an optimal sample size and team members with relatively equal skills and a mix of experience. Our study highlights how low-cost eye-tracking glasses could be an effective tool to analyse some of the human factors for improved medical training taking situational awareness into account as a contributing factor in task and cognitive workload in context. Researching these subjects in a simulation environment would be less expensive, in terms of resources, than trying to conduct this research in hospital settings which would have ethical and other logistical issues.

## 5. Conclusions

In our pilot study of a team having a dedicated team leader as compared to a team without a leader, we could not demonstrate any significant difference in visual attention, performance, behavioural skills, and self-reported workload. Physical demand and thus workload was reported to be significantly higher overall by participants in the group without a team leader and all members of the team felt the need for a dedicated team leader.

## Figures and Tables

**Table 1:** Participants professional training and neonatal experience.

Training Level/Nurse Band (Level)	Neonatal Experience (Years)	Times Completed NLS Course (Number)	Completed NLS within Last 1 Year (Yes/No)	Prior Neonatal Simulation Experiences (Number)	Prior Neonatal Resuscitation Real Life Experiences (Number)
Senior trainee-1	1.5	2	No	6	>30
Senior trainee-2	3	1	No	2	>30
Senior trainee-3	3	2	No	2	>30
Senior trainee-4	2	2	No	6	>30
Senior trainee-5	2.5	2	Yes	6	>30
Senior Nurse practioners-1	20	5	No	>5	>30
Junior trainee-1	1	1	No	1	>20
Junior Nurse practioners-1	2	3	No	<5	3
Junior trainee-2	<1 year	1	No	2	0
Senior Nurse-1	11	2	No	0	4
Senior Nurse-2	7	2	Yes	<5	>10
Senior Nurse-3	9	2	No	<10	20–30

**Table 2:** Visual Fixations and area of interest in participants who were airway operators and wearing eye-tracking glasses.

	Participant 1		Participant 2		Participant 3		
	No Team Leader	Team Leader	No Team Leader	Team Leader	No Team Leader	Team Leader	Wilcoxon Sign Rank Test (*p* Value)
Number of Fixations							
Manikin	51	27	43	23	39	30	0.109
Colleague	23	20	19	10	20	12	0.109
Monitors	38	34	29	16	42	39	0.109
Fixation Time in seconds on specific AOI (=dwell time)							
Manikin	227	151	244	354	218	155	1.000
Colleague	42	34	50	18	53	15	0.109
Monitors	104	279	110	22	291	188	1.000

**Table 3:** NRPE and BAT scores.

	Assessor-1			Assessor-2		
	Team Leader	No Team Leader	Comparison within Groups *p* Value (Paired *t* Test)	Team Leader	No Team Leader	Comparison within Groups *p* Value (Paired *t* Test)
NRPE Decision	100 ± 0	100 ± 0	NA	81.9 ± 3.290	100 ± 0	0.010
NRPE Technique scores	95.3 ± 4.04	93.33 ± 0.577	0.48	98 ± 3.46	90.33 ± 2.3	0.148
NRPE total scores	96.67 ± 2.89	95 ± 0	0.423	94 ± 1.73	93 ± 1.73	0.667
BAT Scores	40.33 ± 3.21	39.66 ± 3.05	0.85	34.67 ± 8.144	34.66 ± 5.03	1.00
All the values are reported in Mean ± standard deviation						

**Table 4:** NASA-TASK LOAD INDEX-Self reporting.

	Team Leader	No Team Leader	Wilcoxon Sign Rank Test (*p* Value)
**All Participants (TL = 12; No TL = 9)**			
Mental Demand	12.5 (9.5–16.5)	15 (14–17)	0.211
Physical demand	5 (2.5–9)	10 (5–12)	0.039
Temporal demand	9.5 (5–15.5)	14 (5–16)	0.898
Performance	5 (4–8)	7 (5–10)	0.543
Effort	10.5 (5–15)	13 (5–15)	0.637
Frustration level	3.5 (1.5–7.5)	5 (4–12)	0.508
**Nurse (TL = 3; No TL = 3)**			
Mental Demand	10 (10–16)	15 (14–18)	0.500
Physical demand	9 (2–10)	10 (3–16)	0.500
Temporal demand	16 (4–16)	16 (14–16)	1.000
Performance	13 (5–14)	9 (5–14)	1.000
Effort	11 (10–13)	15 (13–16)	0.250
Frustration level	15 (3–16)	9 (5–12)	0.750
**Junior trainee (TL = 5; No TL = 3)**			
Mental Demand	14 (9–14)	16 (13–17)	0.75
Physical demand	5 (2–5)	9 (4–12)	0.250
Temporal demand	8 (5–9)	5 (3–16)	1.000
Performance	5 (2–6)	3 (2–7)	0.750
Effort	9 (4–16)	5 (2–12)	1.000
Frustration level	2 (1–4)	4 (3–17)	0.500
**Senior trainee (TL = 4; No TL = 3)**			
Mental Demand	14 (8–17.5)	15 (9–17)	1.00
Physical demand	5 (4–7)	11 (5–15)	0.500
Temporal demand	12 (7.5–14.5)	9 (5–15)	0.750
Performance	4.5 (4–5)	10 (5–16)	0.250
Effort	10 (5–15)	14 (5–16)	1.000
Frustration level	3.5 (1.5–7.5)	4 (1–17)	0.750
All the domains were score from 0–20, with 20 being the worst and ‘0’ being the best			
All the data presented in Median with Inter-quartile range.			

## Data Availability

Deidentified individual participant data will be made available, in addition to study protocols, the statistical analysis plan, and the consent form. The data will be made available upon publication to researchers who provide a methodologically sound proposal and research ethics board approval. Proposals should be submitted to pkannanloganathan@nhs.net.

## Supplementary Materials

The following supporting information can be downloaded at https://www.mdpi.com/article/10.3390/children11081023/s1, Table S1: NRPE and BAT scores for each scenarios by two assessors; Table S2: NASA-TASK LOAD INDEX-Self reporting in participants who were airway operators and wearing eye-tracking glasses; Table S3: Individual NASA-TASK LOAD INDEX scores (after excluding airway operators); Supplementary File S1: Study protocol; Supplementary File S2: Preterm resuscitation scenario, Term resuscitation scenario, Study algorithm and sequence of simulation and team allocation, Modified the NRPE tool, Behaviour Assessment Tool, National Aeronautics and Space Administration Task Load Index, Participant Information Sheet and consent form; Supplementary Figure S1: Study algorithm; Supplementary Figure S2: Image of volunteer performing pre-simulation test for familiarity and calibration; Supplementary data File: Data on the number of fixations and fixation time for each participant and scenario (Excel file).
