# Supplementary material for: Assessment of Visual Attention in Teams with or without Dedicated Team Leaders: A Neonatal Simulation-Based Pilot Randomised Cross-Over Trial Utilising Low-Cost Eye-Tracking Technology
Source: Children (Basel). 2024 Aug 21;11(8):1023. doi: 10.3390/children11081023 (PMC11352304; doi:10.3390/children11081023)
Supplement: Supplementary file 1 [file children-11-01023-s001.zip › children-3143164-Supplemental file 2.pdf]

Assessment of visual attention in teams with or without dedicated team leaders: A neonatal simulation-based pilot study utilizing low-cost eye-tracking technology.

Investigators: Prakash Kannan Loganathan [1,2,3], Anip Garg[1], Robert McNicol[4], Conor Wall [4], Matthew Pointon[4], Peter McMeekin[5], Alan Godfrey[4], Micheal Wagner [6] , Charles Roehr[7,8,9]

Affiliations:

1. Neonatal Intensive care unit, The James Cook University Hospital, Middlesbrough, United Kingdom
2. Clinical Academic Office, Faculty of Medical Sciences, Newcastle University
3. Department of Physics, University of Durham, Durham, United Kingdom.
4. Department of Computer and Information Sciences, Northumbria University, Newcastle upon Tyne, UK
5. Department of Nursing, Midwifery, and Health, Northumbria University, Newcastle upon Tyne, UK
6. Division of Neonatology, Pediatric Intensive Care and Neuropediatrics, Department of Pediatrics, Comprehensive Center for Pediatrics, Medical University of Vienna, Vienna, Austria
7. Nuffield Department of Population Health, Medical Sciences Division, National Perinatal Epidemiology Unit, University of Oxford, Oxford, UK
8. Newborn Services, Southmead Hospital, North Bristol Trust, Bristol, UK.
9. Faculty of Health Sciences, University of Bristol, Bristol, UK.

Address for Communication:

Prakash Kannan Loganathan (ORCID: 0000-0003-3717-8569)

James Cook University Hospital

Marton Road, Middlesbrough TS4 3BW

Email: pkannanloganathan@nhs.net

Phone: 01642 854874

Fax: 01642854488

Key words: Human Factors, neonatal resuscitation, visual attention, eye tracking, team performance

Funding: Nil

Financial Disclosure: The authors have no financial relationships relevant to this article to disclose.

Conflicts of Interest: Other authors have no conflicts of interest relevant to this article to disclose.

Word count: Abstract 200; Manuscript: 3407

Table of contents

Appendix- A	Preterm resuscitation scenario
Appendix B	Term resuscitation scenario
Appendix C	Study algorithm and sequence of simulation and team allocation
Appendix D	Modified the NRPE tool
Appendix E	Behaviour Assessment Tool
Appendix F	National Aeronautics and Space Administration Task Load Index
Appendix G	Participant Information Sheet and consent form
Supplemental Figure 1	Image of eye tracking glasses
Supplemental Video 1	Video of eye tracking glasses

Appendix - A

HUFIN_Pretrem scenario

Setting	NNU (Delivery Suite scenario)
Scenario	Management at pre-term delivery

Learning objectives

- This simulation is for research purpose and learning, not for assessment. Confidential.
- Consent form
- Low fidelity doll, please perform action and ask for the response.
- If the team has not used resuscitaire then we would go through this for few mins

1. Appropriate set up of resuscitaire + equipment, choice of mask/pressures/airway adjunct sizing/intubation sizing
2. NLS algorithm- Focusing on good airway management; neutral position, c-shaped seal, two-person technique/jaw thrust.
3. Early recognition of need for intubation ± surfactant ± pneumothorax
4. Communication between the team.

Background and equipment

Background:
Being a tier 3 NNU pre-term deliveries are expected and common and we should all be familiar with setting up and checking a resuscitate as well as knowing basic management and stabilisation of a pre-term neonate prior to transfer to NICU.

Relevant areas of the RCPCH curriculum:

1. Domain 2 Professional skills: communication
2. Domain 3 Professional skills: procedures
3. Domain 4 Professional skills: Patient management
4. Domain 6 Leadership and team working

Scenario: Preterm delivery

Assistants:
- Staff nurse
- SHO/middle grade
- NICU senior registrar.

Equipment:
- Mannequin (Peretrem)-Anne/Nenasim
- Resuscitaire with oxygen/air connection
- Resus equipment- Plastic bag, Neohelp, woolen hat, preterm/term masks selection, suction, ET tubes, laryngoscope, laryngoscope blades, pulse oximeter with sensor, thermometer, stethoscope, Three way taps, butterfly needle.
- Two IPADS

Briefing for candidate

1. **Scene-setting:**

Background: You are asked to attend the delivery suite. Primip mother, 23 years old presented in active labour 30 minutes ago. She has not been given antenatal steroids. Pregnancy was low risk, serology has been negative. She is 25weeks gestation

2. **Assistance:** A nurse/SHO/registrar will be present during the scenario. They will act on your instruction but not volunteer information that is not requested. If other appropriate help is needed at any stage, ask for it.

3. The scenario will run until a natural conclusion (approximately 15-20 minutes), and thereafter we will discuss the scenario as a group.

- All team members would be present during the team briefing
- Team huddle for few mins (5mins)

Scenario flowchart

INITIAL PLAN

1) Check resuscitaire: Manual mode. Choose appropriate mask, check pressure (need to set 25/5), have rosti bag, woolen hat, pulse oximeter, check laryngoscope light, blades of different sizes, ET tubes 2.53mm, emergency trolley, thermometer, stethoscope

3) Baby born and brought to resuscitaire- start timer

4) Place in rosti bag

ASSESSMENT

Blue, floppy, HR 80bpm, no gasp/ respiratory effort —>Call for senior support if not already done

Team may consider DCC

A: Position, 5x inflation breaths-> no chest wall rise
Re-position—> no chest rise UNTIL 2 person technique
Once chest wall rise re-assess
HR>100, pink centrally, improved tone, no respirations
If sats probe applied to right hand saturations 30-40% (Can titrate o2)
30 seconds ventilation breaths
HR>100, pink centrally, fair tone, irregular resps

EXPECTED ACTIONS

- Assess tone, colour, respiration, HR
- Airway position+ 2 person technique/airway adjunct
- Inflation/ventilation breaths
- apply sats probe to right hand
- Titrate O_2 to improve SpO_2
- Consider need for intubation
- Ask for senior help

MANAGEMENT

Senior registrar attends
Titrate oxygen
Once Fi02 titrated, saturations do not reach more than 70%.
Intubate with size 2.5mm. Use calorimeter and assess.
Surfactant administration.
If intubated to note sats increase.

IF INTUBATES IMMEDIATELY

- Calorimeter change , chest AE, sats>92%
- After stabilising ET tube for sats to drop to 50s, requiring 100% Fi02
- U/L chest preominance

Re-assessment
No AE right lung, AE left lung

EXPECTED ACTIONS

- Senior help
- Intubation when available
- Stabilisation
- Transfer to NICU
- Update parents

EXPECTED OUTCOME

- Plan transfer to NICU +/- light, chest needling, CXR
- Update parents

RESOLUTION

- Stabilise and further management on NICU- surfactant + CXR.

Appendix B

HUFIN_Term scenario

Resuscitation following Maternal abruption

TARGET:

1. SHO/ANNP
2. Nurses
3. Registrars

BACKGROUND:

This scenario fulfils covers many RCPCH curriculum requirements.
Not an uncommon scenario.
Useful for newly started trainees in the NICU.

RELEVANT AREAS OF THE CURRICULUM

RCPCH curriculum

1. Domain 2 Professional skills: communication

2. Domain 3 Professional skills: procedures

3. Domain 4 Professional skills: Patient management

INFORMATION FOR FACULTY

LEARNING OBJECTIVES

These have been divided into bullet pointed lists

Generic

- History
- Assessment
- Communication

Scenario-based

- Neonatal resuscitation
- Procedure- Intubation, UVC insertion

SCENE SETTING

Mannequin-Low fidelity term mannequin.

Environment- Operation Theatre

BRIEF SCENARIO SUMMARY

Attending Category 1 LSCS for suspected abruption of placenta.

TIMINGS

Estimated scenario run time:
20 mins

Estimated debrief time: 20
mins

EQUIPMENT AND CONSUMABLES

Monitor-Spo2, HR, BP
Pulse oximeter sensor
ET tube, Laryngoscope, Pedicap, Meconium aspirator
Suction, towels, Hat
Suction catheter
Blood gas result
Resusctaire, Tom thumb, term mask
UVC Catheter, Saline, Adrenaline, syringes, Blood (ink)
Stethoscope

PERSONNEL

Nurse A
Nurse B
ANNP/SHO
Registrar

ADDITIONAL RESOURCES

None

MATERNAL/NEONATAL HISTORY

Day shift team: Registrar & SHO/ANNP

Background: Called to attend a Category 1 C section under GA happening in theatre 24 (indication- suspected abruption

of placenta with foetal bradycardia and sinusoidal pattern on CTG). Estimated foetal weight is 3000 gm.

Antenatal History: 21year, Primigravida mother presented with decreased FM and blood tinged amniotic fluid. Pregnancy (38 weeks currently) was healthy and serologies all were negative with uneventful pregnancy so far.

Birth & Resuscitation: You can gather your team quickly. Its 12 am and consultant is at home. There is a registrar and SHO available along with 1 admission nurse.

Team reaches theatre 24. Expected delivery in next 5 minutes

Expected actions:
Pre brief /Huddle with delegation of roles
Equipment checks including resuscitaire
Consultant aware of the situation

ADDITIONAL INFORMATION

Nil

VOICE OF THE PARENTS (IF REQUIRED)

Not required

VOICE OF THE TELEPHONE HELP BRIEFING (IF REQUIRED)

If help is available over the telephone, then this section explains what that conversation would entail. This may not be necessary for every scenario

CONDUCT OF THE SCENARIO

Sim scenario starts here

↓

0010 hr
Baby born
No cry, very pale and floppy
Covered with blood
HR Nil, No respiratory efforts
Baby arrives at resuscitaire at 20 seconds

Expected actions:
Start clock at birth
Drying, discarding wet towel and wrapping of baby with warm towels, Hat on, spO2 probe applied (preductal)
Start Inflation breaths-→ no chest rise

2nd set of inflation breaths (with two-person technique)-→ minimal/no chest rise

Expected actions:
Call for extra help (Consultant)
Prepare for intubation.

(Blood clots seen during suction of mouth during intubation)

0012 hr (2 minutes)
Poor chest rise following intubation, no colour change in Pedicap
HR- not recordable, Pale, no respiratory efforts

Expected actions:
Check bilateral air entry, and ET length at upper lips, Use meconium aspirator rather than repeated attempts at intubation- There could be many actions at this point: Suction through ET tube, Increasing pressure, check for pneumothorax

If uses meconium aspirator→chest rise improves with colour change in the Pedicap
HR 40/min, no respiratory efforts, spO2- unrecordable

Expected actions:
Continue ventilation breaths for 30 seconds and reassess

After 30 seconds, (3 minutes)
HR 40/min, no respiratory efforts, pale and floppy, spO2 unrecordable

Expected action:
Start chest compression- Fio2 to 100%
Ask team for prepare for drugs and UVC access
Ask for emergency blood

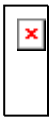

After 30 seconds, (3 minutes, 30 seconds)
HR 40/min, No respiratory efforts, spO2 unrecordable

Expected action:
Continue chest compression
FiO2 to 100%
UVC secured

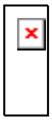

After 30 seconds (4 minutes)
HR 50/min, No respiratory efforts, spO2 40%

Expected actions:
Continue chest compression
First dose of Adrenaline given followed by flush- 20 micrograms kg^{-1} (0.2 mL kg^{-1} of 1:10,000 **adrenaline**

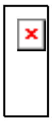

After 30 seconds (4 minutes 30 seconds)
HR 50/min, no respiratory efforts, spO2 65%

Expected actions:
Continue chest compression
Continue ventilation breaths
Second dose of adrenaline
Administer Saline bolus 10ml/kg
Ask for blood

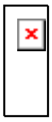

After 30 seconds (5 minutes)
HR 60/min, no respiratory efforts, spO2 70%

Expected actions:
Discontinue chest compression, continue ventilation breaths

After 30 seconds (5 minutes 30 seconds)
HR 80/min, minimal gasping efforts, spO2 80%
Blood arrives

Expected actions:
Start Blood transfusion 20 ml/kg
Continue ventilation breaths
Secure ET by Neobar, insert an NG tube and aspirate

After 30 seconds (6 minutes)
HR 110/min, spO2 92%, gasping respiration

Expected actions:
Continue ventilation breaths
Makes arrangement to transfer to the unit

End of scenario

Expected actions
Nurse:

1. Checking equipment
2. Saturation probe and hat
3. Temperature checks
4. Help with intubation and UVC catheterisation
5. Prompt to ask for extra help if not sought timely by registrar/SHO

Registrar & SHO

- Intubation
- Recognise tracheal obstruction
- Ask for saline bolus and emergency blood
- Ask for help from consultant
- Ask for cord gases
- SBAR handover to the consultant
- Update parents

SCENARIO TITLE

INFORMATION FOR PARTICIPANTS

KEY LEARNING POINTS

1. Recognise tracheal obstruction early
2. Suspected volume loss and consider replacement in abruption code

5. Consider for need of intubation after use of 2-person technique/Airway adjunct after 2nd set of inflation breaths in case of no/poor chest rise and very slow HR and unrecordable saturations *

6. Use of meconium aspirator rather than repeated intubations and use of suction catheter after poor chest rise following intubation *

7. Recognizes volume loss– ask for normal saline bolus after adrenaline before blood transfusion*

Appendix - C

Time	Action	
0745-0815	Equipment and set up checking	Team
0830-0930: Simulation 1<br><br>Team 1<br><br>PRETERM<br><br>NO TEAM LEADER	- 10 mins for set up<br>- 5 mins for the participants briefing<br>- 5 mins for team huddle<br>- 20 mins for simulation<br>- 10 mins for debrief<br>- 10 min break	-Junior medical staff<br>-Senior medical staff-1<br>-Neonatal nurse
0930-1030: Simulation 2<br><br>Team-1<br><br>TERM<br><br>TEAM LEADER	- 10 mins for set up<br>- 5 mins for the participants briefing<br>- 5 mins for huddle<br>- 20 mins for simulation<br>- 10 mins for debrief<br>- 10 min break	- Junior medical staff<br>- Senior medical staff-1<br>- Senior medical staff-2 (TEAM LEADER)<br>- Neonatal nurse
1030-1100: coffee break		
1100-1200: Simulation 3<br><br>Team-2<br><br>PRETERM<br><br>TEAM LEADER	- 10 mins for set up<br>- 5 mins for the participants briefing<br>- 5 mins for team huddle<br>- 20 mins for simulation<br>- 10 mins for debrief<br>- 10 min break	- Junior medical staff<br>- Senior medical staff-1<br>- Senior medical staff-2 (TEAM LEADER)<br>- Neonatal nurse

1200-1300: Simulation 4<br><br>Team-2<br><br>TERM<br><br>NO TERM LEADER	- 10 mins for set up<br>- 5 mins for the participants briefing<br>- 5 mins for team huddle<br>- 20 mins for simulation<br>- 10 mins for debrief<br>- 10 min break	- Junior medical staff<br>- Senior medical staff-1<br>- Neonatal nurse
1300-1345	Lunch	
1345-1400	Checking up	
1400-1500: Simulation 5<br>Team-3<br><br>TEAM LEADER<br><br>PRETERM	- 10 mins for set up<br>- 5 mins for the participants briefing<br>- 5 mins for team huddle<br>- 20 mins for simulation<br>- 10 mins for debrief<br>- 10 min break	- Junior medical staff<br>- Senior medical staff-1<br>- Senior medical staff-2 (TEAM LEADER)<br>- Neonatal nurse
1500-1600: Simulation 6<br><br>Team-3<br><br>No TEAM LEADER<br><br>TERM	- 10 mins for set up<br>- 5 mins for the participants briefing<br>- 5 mins for team huddle<br>- 20 mins for simulation<br>- 10 mins for debrief<br>- 10 min break	- Junior medical staff<br>-Senior medical staff-1<br>-Neonatal nurse
1600-1630	Organising group wrap up	

Appendix -D

- **A**ssessor Name:
- Simulation no:
- Scenario: Preterm /Term
- Did your scenario had a dedicated team leader: Yes / No
- Team members:

Modified Neonatal Resuscitation Performance Evaluation

Decision: Y: (Indicated / Done, Not indicated / Not Done), N: (Not Indicated / Done, Indicated / Not Done)<br>Y: Yes (1 point), I: Incomplete (0 points), N: No (0 points).		Y	I	N	NA
**Decision**	**Initial Steps**<br>*Asks pre-brief questions*<br>*Assigns tasks / Calls for help if needed*<br>*Select and check appropriate equipment*<br>Start clock				

**Technique**	Dries, stimulates appropriately / Removes wet linen. Apply hat<br><br>Preterm: apply NEOHELP/NEOWRAP bag, apply hat<br><br>Turn on the radiant heating				
	Position in neutral position				
**Decision**	**Assess colour, tone, heart rate and respiration**<br><br>**Proceed to First set of Inflation Breath**<br>*Apnea / gasping/ No respiratory effort*<br>*HR < 100*				
**Technique**					
	Titrate FiO2 appropriately appropriate for gestational age- Consider placing pulse ox				
	Start inflation breath *5, each for 2-3 seconds				
	Assess Chest Movement & Assess heart rate				
**Decision**	**Second set of inflation breath**<br>*Apnea / gasping/ no respiratory effort/ no chest movement*<br>*HR < 100*				
**Technique**					
	Verify neutral position, mask size, mask leak				
	Start inflation breath *5, each for 2-3 seconds				

	Assess Chest Movement and Assess heart rate				
**Decision**	**Ventilation breath**<br>*Apnea / gasping / no or poor respiratory effort- But chest movement noted.*<br>*HR>100*				
**Technique**					
	Start ventilation breath each for 1-2 seconds for 30 seconds				
	Assess Chest Movement and Assess heart rate				
**Decision**	**Proceed to Alternative Airway Placement**<br>*Apnea / gasping,*<br>*No chest movement or HR < 100 despite two sets of inflation breath*<br>*Prolonged ventilation breaths*				
**Technique**					
	Laryngoscope in left hand, appropriate size laryngoscope blade				
	Correct ETT/LMA size				
	Correct depth				
	Secures ETT/LMA				
	Confirmed placement with pedicap				
**Decision**	**Proceed to Chest Compressions**<br>*HR < 60, not increasing with two sets of inflation breaths*				

**Technique**	30 seconds of ventilation breath before chest compression				
	Hands encircle chest in correct position				
	Depth and recoil appropriate				
	3:1 coordination with ventilation breaths				
	100% FIO2				
**Decision**	**Proceed to Umbilical Venous Catheterization**<br>*IV medications/volume or blood indicated*				
**Technique**	Equipment Prep: correct size (F) single lumen UVC, stopcock, flush				
	Patient Prep: prep skin, umbilical tie, cut cord				
	Placement: correct depth, blood return, holds catheter until secured				
**Decision**	**Proceed to Epinephrine**<br>*HR < 60 with 30 sec chest compressions + ventilation breath*				
**Technique**	Dose 1: Correct dose (20 micrograms/ kg (0.2 mL/ kg of 1:10,000 adrenaline) + flush				
	Dose 2: Correct dose (20 micrograms/ kg (0.2 mL/ kg of 1:10,000 adrenaline) + flush				
**Decision**	**Proceed to Volume**<br>*Unresponsive to epinephrine AND history or symptoms suggestive of hypovolemic shock*				
**Technique**	Correct dose and volume: Normal Saline:10ml/kg; O negative blood: 10-20ml/kg				

Compliance Score Summary

	Y: Yes (1 point), I: Incomplete (0 points), N: No (0 points), All times in seconds<br>If Section was N/A enter NA in blank
**Initial Steps**	Points Possible Score<br>Decision _____ / 1 x 100% = _____ %<br>Technique _____ / 2 x 100% = _____ %<br><br>Total _____ / 3 x 100% = _____ %
**First set of inflation breath**	Decision _____ / 1 x 100% = _____ %<br>Technique _____ / 3 x 100% = _____ %<br><br>Total _____ / 4 x 100% = _____ %
**Second set of inflation breath**	Decision _____ / 1 x 100% = _____ %<br>Technique _____ / 3 x 100% = _____ %<br><br>Total _____ / 4 x 100% = _____ %
**Ventilation breath**	Decision _____ /1 x 100% = _____ %<br>Technique _____ / 2 x 100% = _____ %<br><br>Total _____ / 3 x 100% = _____ %

**Alternative Airway Placement**	Decision _____ / 1 x 100% = _____ %<br>Technique _____ / 5_ x 100% = _____ %<br><br>Total _____ / 6 x 100% = _____ %
**Chest Compressions**	Decision _____ / 1 x 100% = _____ %<br>Technique _____ / 5 x 100% = _____ %<br><br>Total _____ / 6 x 100% = _____ %
**Umbilical Venous Catheter Placement**	Decision _____ / 1 x 100% = _____ %<br>Technique _____ / 3 x 100% = _____ %<br><br>Total _____ / 4 x 100% = _____ %
**Epinephrine**	Decision _____ / 1 x 100% = _____ %<br>Technique _____ / 1 x 100% = _____ %<br><br>Total _____ / 2 x 100% = _____ %
**Volume**	Decision _____ / 1 x 100% = _____ %<br>Technique _____ / 1 x 100% = _____ %<br><br>Total _____ / 2 x 100% = _____ %
**Total**	Decision _____ / ___ x 100% = _____ %<br>Technique _____ / _____ X 100% = _____ %<br><br>Total _____ / _____ x 100% = _____ %

Appendix - E

- **A**ssessor Name:
- Simulation no:
- Scenario: Preterm /Term
- Did your scenario had a dedicated team leader: Yes / No
- Team members:

NEONATAL RESUSCITATION
BEHAVIORAL PERFORMANCE EVALUATION

Ten behavioral markers of individual and team performance during neonatal resuscitation are identified below. For each marker circle the number that best describes the level of performance displayed. PLEASE DO NOT CIRCLE TWO RESPONSES!

0 = poor **2 = acceptable** **4 = excellent**

1. Knowledge of the Environment

0 1 2 3 4

Appears disoriented; is uncertain as to layout of delivery room and location of equipment such as intubation module and crash cart; fails to insure working condition of all equipment, including laryngoscope and endotracheal tubes; fails to ask questions of others in the environment.	Appears comfortable with environment; knows where equipment/supplies reside; checks the equipment as time allows; if unable to locate equipment/supplies; asks questions of others in the environment as needed.	Knows all aspects of environment; thoroughly checks all equipment to insure that it is present and in working order prior to delivery; confirms readiness of environment with members of team; does not hesitate to ask questions of others in the environment when the need arises.

2. Anticipation of and Planning for Potential Problems

0 1 2 3 4

Does not appear prepared for the delivery; does not inquire as to why the presence of the pediatric team is requested at the delivery; does not inquire as to the number of fetuses, gestational age, and presence of meconium; does not assign roles to each team member; does not recognize situations where the composition of the team is inadequate (in number and/or expertise) and takes no steps to remedy this situation; fails to react to changing circumstances as the case evolves.	Appears prepared for the delivery; inquires about one or two of the following: number of fetuses, gestational age, or presence of meconium; recognizes whether all of the appropriate personnel are present and initiates a plan to contact those who are not; implicitly assigns the roles of the team members (by positioning around bedside, etc.) but does not explicitly assign tasks; recognizes changing circumstances and begins to devise strategies to deal with them.	Appears thoroughly prepared for the delivery; inquires as to why the presence of the pediatric team is requested at the delivery; inquires about all three of the following: number of fetuses, gestational age, or presence of meconium; recognizes prior to the delivery whether all of the appropriate personnel are present and actively ensures that the team is complete by the time the baby is born; explicitly assigns the roles of the team members prior to delivery (conducts a briefing); asks questions indicating an in-depth understanding of potential problems and subsequent consequences of the evolving case; does not appear surprised by predictable situations; effectively deals with changing circumstances.

3. Assumption of Leadership Role

0 1 2 3 4

Team members appears nervous, "rattled", uncomfortable; no one clearly identifies himself/herself as the person in charge; activities of the team are not coordinated; team members tend to stand back and take a "hands-off" approach; no one acts to inspire confidence among team members.	Team members appear calm; a team member implicitly assumes the leadership role but does not explicitly state that he/she is the leader; activities of the team are coordinated; team members take a "hands-on " approach.	Team members appear calm; the leader is clearly identified prior to the delivery; leader helps to facilitate coordination of all of the activities of the team; the leader is actively engaged in the situation and calmly inspires confidence among team members.

4. Communication Among Team Members

0 1 2 3 4

Problems stated in incorrect or confusing terminology; members do not speak clearly; voices are either too soft or too loud; team members are "talked down to"; intended recipients of commands are not clearly identified ("thin air" commands); "repeat-backs"/"closed loop" communications do not occur; team members "talk over" one another.	Problems are communicated to the team; team members speak clearly; tone of voices varies from soft to loud but audible to all team members; attempts to identify the intended recipients of commands are made; "repeat-backs"/"closed loop" communications occur during most communications.	Problems are clearly communicated to the team; team members speak clearly, succinctly, and in even tones; important communications can easily be heard by the other members of the neonatal team; intended recipients of commands are clearly identified; "repeat-backs"/"closed loop" communications occur at all times; team members listen to others, clarify ambiguous communications and encourage cooperation.

5. **Distribution of Workload/Delegation of Responsibility**

0	1	2	3	4

Some team members try to "do it all", failing to recognize the (potential) contributions of others who do little or nothing; some team members are asked to exceed their abilities without receiving appropriate guidance.	Team members implicitly understand what tasks they are to undertaken; tasks appear to be evenly divided among team members; supervision occurs at least intermittently; all necessary tasks are accomplished.	Specific tasks are clearly assigned to specific team members; the skills of all team members are utilized well; over-taxed team members are assisted/relieved when necessary; an appropriate level of supervision is always provided.

6. **Attention Allocation**

0	1	2	3	4

Easily distracted; unable to "tune out" unimportant input such as background noise; becomes caught up in details and fails to see the "big picture"; does not prioritize demands for attention.	Does not become easily distracted; recognizes the "big picture" and is able to tune out the majority of unnecessary details; adequately prioritizes; avoids fixation errors.	Does not become distracted; cognizant of details yet adequately monitors patient's overall condition; prioritizes demands for attention well; avoids fixation errors.

7. **Utilization of Information**

0	1	2	3	4

Does not recognize emergency situations; fails to recognize disease states requiring intervention; ignores part of data in formulating a diagnosis; does not incorporate historical information into approach to patient; unable to reach conclusions despite a reasonable data base; fails to continually reassess; persists in original course of action despite indications to change.	Recognizes emergency situations; recognizes disease states requiring intervention; able to assimilate most data (including historical data) in formulating a diagnosis and plan of care; reassesses patient status as needed; recognizes changes in patient condition.	Recognizes emergency situations instantly; recognizes all disease states requiring intervention in the delivery room; able to quickly assimilate all pertinent data (including historical data) in formulating a diagnosis and logical plan of care; interprets physical findings accurately; repeats physical examination when findings are equivocal; frequently reassesses patient status; immediately recognizes changes in patient condition and revises plan of care accordingly.

8. **Utilization of Resources**

0	1	2	3	4

Fails to recognize the professional skills of others; does not identify potential alternatives when presented with equipment or personnel failures.	Utilizes the expertise of other team members appropriately most of the time; may struggle with equipment or personnel failures but eventually problem-solves for other solutions.	Readily solicits and incorporates the expertise of others without prompting; reacts to equipment or personnel failures by quickly identifying and implementing alternative solutions.

9. **Call for Help Early Enough/ Recognition of Limitations**

0	1	2	3	4

Fails to recognize own limitations; does not request assistance when needed; recognizes own limitations but attempts to exceed these limitations, thereby placing patient at risk; calls for help when not indicated.	Recognizes own limitations in content knowledge and technical and behavioral skills; calls for help once these limitations are acknowledged.	Immediately recognizes when at his/her limits in medical knowledge and technical and behavioral skills; recognizes situations where additional help will be required and requests such assistance early (before it is actually needed).

10. Professional Behavior/Interpersonal Skills

0	1	2	3	4

Engages in unnecessary conversation; makes inappropriate comments; uses profanity; shows little concern for patient's/family's comfort; approach to family members is demeaning, abrupt, clumsy or otherwise inappropriate; is unable to give or take advice gracefully; easily becomes defensive; lacks communication skills.	Keeps unnecessary conversation to a minimum; language and approach are professional most of the time; regards family with a caring attitude; recognizes and responds appropriately to the majority of nonverbal and verbal cues; accepts advice without complaint or defensiveness; supervises and teaches in a non-threatening manner.	Maintains composure at all times; does not engage in any unnecessary conversation; language and approach are always professional; consistently demonstrates a caring attitude toward patients and families; recognizes and responds to all nonverbal and verbal cues; readily accepts advice and encourages input from other team members; generates a positive atmosphere while supervising and teaching; non-judgmental; non-defensive.

11. Overall Behavioral Skills Rating

0	1	2	3	4
Multiple major deviations from standard of care, at least one of which is potentially fatal; overall negative effect on patient; poor.	Few major deviations from standard of care, none of which is potentially fatal; sub-par, below average, inadequate.	No major deviations from standard of care; minor deviations present, but overall care of patient acceptable; average, adequate.	Few minor deviations from standard of care, no negative effects on patient; above average, good.	No major or minor deviations from standard of care; excellent, exemplary.

Comments:

Total score (add score from 1-11): ________

% Score (total score / 44): ________

Appendix · F

Assessment of workload using NASA-TASK LOAD INDEX (NASA-TLX)

(Please encircle your responses)

- Your role: Nurse /Tier-1/ Tier -2
- How many years of experience do you in working with neonates: <2year/ 3-5 years/ >5 years.
- Scenario: Preterm /Term
- Did your scenario had a dedicated team leader: Yes / No

Please read the following definition before providing your response on Page2

RATING SCALE DEFINITIONS		
Title	Endpoints	Descriptions
MENTAL DEMAND	*Low/High*	How much mental and perceptual activity was required (e.g., thinking, deciding, calculating, remembering, looking, searching, etc.)? Was the task easy or demanding, simple or complex, exacting or forgiving?
PHYSICAL DEMAND	*Low/High*	How much physical activity was required (e.g., pushing, pulling, turning, controlling, activating, etc.)? Was the task easy or demanding, slow or brisk, slack or strenuous, restful or laborious?
TEMPORAL DEMAND	*Low/High*	How much time pressure did you feel due to the rate or pace at which the tasks or task elements occurred? Was the pace slow and leisurely or rapid and frantic?
EFFORT	*Low/High*	How hard did you have to work (mentally and physically) to accomplish your level of performance?
PERFORMANCE	*Good/Poor*	How successful do you think you were in accomplishing the goals of the task set by the experimenter (or yourself)? How satisfied were you with your performance in accomplishing these goals?
FRUSTRATION LEVEL	*Low/High*	How insecure, discouraged, irritated, stressed and annoyed versus secure, gratified, content, relaxed and complacent did you feel during the task?

Please mark your feeling in the scale of 1 to 20, in each domain. 20 being the worst experience and 1 being the best experience

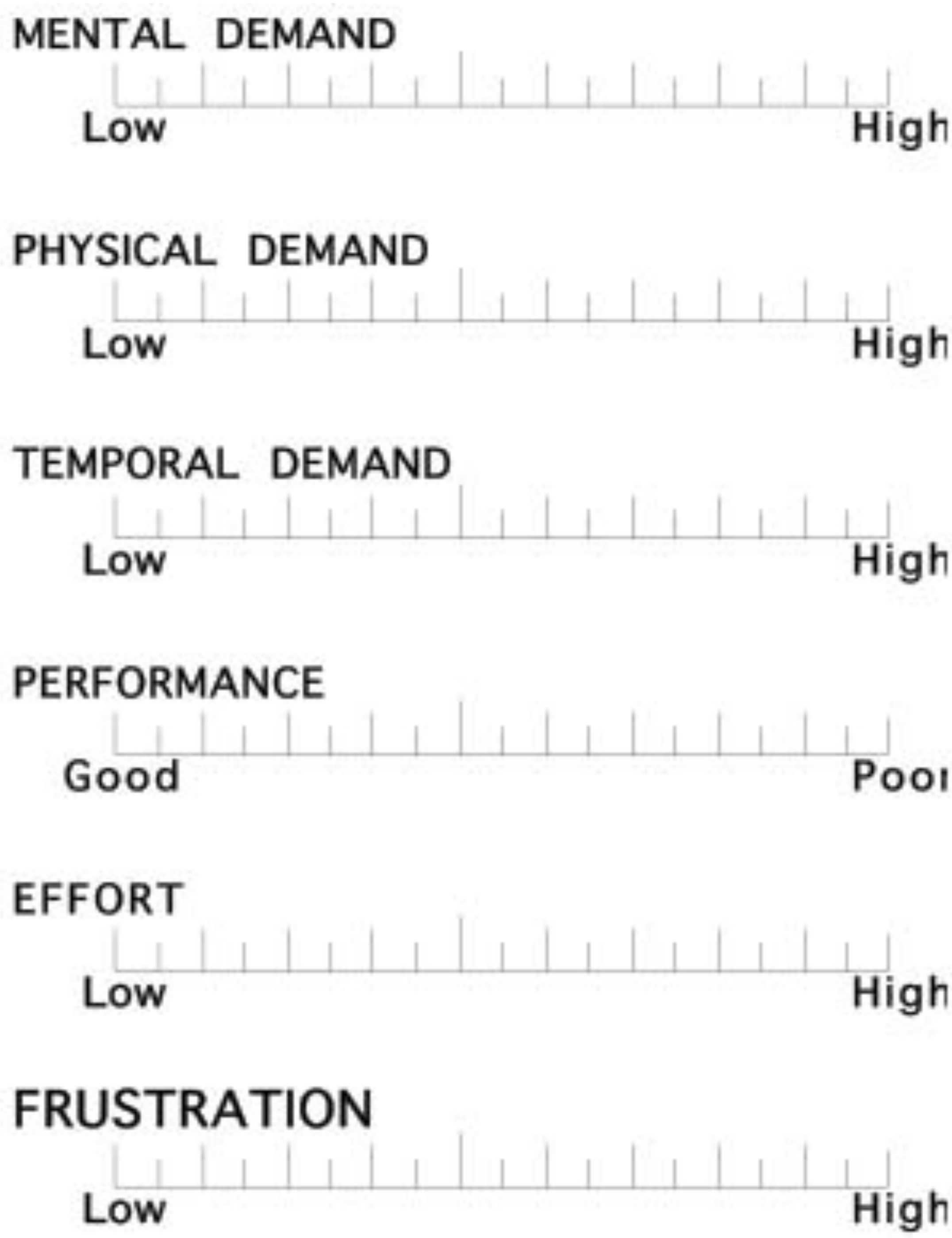

Eye tracking glasses:

- distracted me during the whole scenario: Yes / No / Sometimes-Maybe
- felt uncomfortable: Yes / No / Sometimes-Maybe
- I would wear eye tracking glasses during a real scenario: Yes / No / Sometimes-Maybe

Appendix - G

Title: HUman Factors In Neonatal resuscitation (HUFIN): A simulation-based pilot observational study

Investigator(s): Prakash Kannan Loganathan[1*], Michael Wagner, Robert McNicol[2], Matthew Pointon[2], Peter McMeekin[3], Alan Godfrey[2]

This consent form, a copy of which has been given to you, is only part of the process of informed consent. It should give you the basic idea of what the research is about and what your participation will involve. If you would like more detail about something mentioned here, or information not included here, you should feel free to ask. Please take the time to read this carefully and to understand any accompanying information.

BACKGROUND

Newborn infants sometimes need help with their breathing at the time of birth. This is called resuscitation. In United Kingdom, we follow structured steps to help babies at birth. Often, the main person helping the baby's breathing will be performing other tasks like leading the team, communicating with other team members, and monitoring other team member's performance. Also, most teams don't follow standardized briefing/checklist before helping the babies.

Doing multiple roles at the time of helping babies at birth could lead to reduced focus on the task and possibly affect the outcome. These factors which are based on human behaviour are called as "Human factors". Many factors can affect the success or failure of helping the babies at birth. Human factors could be one among them.

We are interested to find out, whether following structured checklist and having separate dedicated team leader, so that person helping babies breathing don't have to focus on other tasks, would help in these situations?

We are planning to understand this by studying our visual attention or where do people look and what do they pay attention to? This will be done using specialized eye tracking glasses. Using eye-tracking glasses, we can record where the person helping babies breathing is looking, as well as how the team is talking to each other. We were also planning to understand the performance of the team using standardized scoring system. By analyzing this data, we hope to better understand how human factors could affect the success of helping babies at birth.

WHAT IS THE PURPOSE OF THE STUDY?

You are being asked to take part in a simulation research study. The purpose of this study is to assess the situational awareness (how well they keep track of what is happening around them), where they are looking (eye-tracking / visual attention), how they perform during simulated resuscitation.

WHAT WOULD I HAVE TO DO?

We are asking you to volunteer your time for this study. We anticipate that the study will take approximately 60 minutes of your time. We will schedule sessions to occur during the day, that is convenient to you.

If you agree to participate in this study, we will collect basic information your neonatal experience like your role (Nursing experience, level of Paediatric training). You will be asked to assist in 2 simulated neonatal resuscitation scenarios. You will be oriented to the simulation lab and fitted with eye tracking glasses. Then, you will be asked to participate in two different resuscitation scenarios. You will be playing usual role during the simulation. For example, if you are senior doctor, you will be playing the same role during the study. For both simulations, you will be expected to perform using NLS, UK algorithm.

The entire scenarios will be video recorded using a camera placed in the simulation room. Separate videos will be recorded using person helping with babies breathing and wearing eye tracking glasses. This video will provide the video from the person's viewpoint, where the person is looking, how long the person looking. At the end of the study, we will ask you to complete very short questionnaire/feedback, which should not take no longer than two minutes.

Most importantly, participation is completely voluntary, and your performance during the simulation will not be disclosed to your supervisor, other team members or department head. Video recordings will be used only to analyze responses and to analyze resuscitation steps and timing, will be deleted after 5 years.

WHAT ARE THE RISKS?

There are no specific risks associated with participating in this study.

WHAT ARE THE BENEFITS?

The study is designed to assess situational awareness during simulated neonatal resuscitation. If you agree to participate, you will be helping us to understand the situational awareness during simulated neonatal resuscitation of health care providers like yourself.

DO I HAVE TO PARTICIPATE?

Your participation is entirely voluntary. If you decide to withdraw, this can be done at any time before, during, or immediately after the simulations. The final time point where participation can be withdrawn is at the start of data analysis. Any recorded data or survey results will be immediately destroyed.

WILL WE BE PAID FOR PARTICIPATING, OR DO WE HAVE TO PAY FOR ANYTHING?

You will not be paid for participating. You do not have to pay for anything.

WILL MY RECORDS BE KEPT PRIVATE?

Your participation in the study will be treated as confidential. You will be identifiable on the videos, but we will not collect any other personally identifiable information. Further, all videos are encrypted. Only the researchers will have access to the data, which will be stored in password-protected, encrypted computers. All the data will be stored under lock and key in the researchers' office. Once data analysis has been completed, all information will be presented in summary format, thereby making it impossible to identify individual participants. The data will be destroyed five years after completion of the study.

SIGNATURES

Your signature on this form indicates that you have understood to your satisfaction the information regarding participation in the research project and agree to participate as a subject. In no way does this waive your legal rights nor release the investigators or involved institutions from their legal and professional responsibilities. You are free to withdraw from the study at any time without jeopardizing your future employment. If you have further questions concerning matters related to this research, please contact: Dr. Prakash Loganathan, email: pkannanloganathan@nhs.net
Tel: 07481492632

If you have any questions concerning your rights as a possible participant in this research, please contact the Research & Development, South Tees NHS foundation trust at stees.dtvra@nhs.net or Phone: 01654 854089

You can find out more about how we use your information by contacting one of the members of research team using the contact information below or by reviewing www.hra.nhs.uk/information-about-patients/. You can also contact the Sponsor's Data Protection Officer at steven.orley@nhs.net or 01642 50850. Further information with regards to data protection can be accessed at **www.hra.nhs.uk/patientdataandresearch**

Non-identifiable Information about you

Paediatric Training	
Nurse band (level)	
Neonatal experience (years)	
Times completed NLS course (number)	
Completed NLS within last 1 year (yes/no)	
Prior neonatal simulation experiences (number) in the last 1 year	
Prior neonatal resuscitation real life experiences (number) in the last 1 year	

Northumbria University NEWCASTLE

South Tees Hospitals NHS Foundation Trust

MEDIZINISCHE UNIVERSITÄT WIEN

Title of Project: HUman Factors In Neonatal resuscitation (HUFIN): A simulation-based pilot observational study

Principal Investigator(s): Dr. Prakash Loganathan

Phone Number(s): 07481492632

	Yes	No
Do you understand that you have been asked to be in a research study?	☐	☐
Have you read and received a copy of the attached Information Sheet?	☐	☐
Do you understand the benefits and risks involved in taking part in this research study?	☐	☐
Have you had an opportunity to ask questions and discuss this study?	☐	☐
Do you understand that you are free to withdraw from the study at any time, without having to give a reason and without affecting your employment?	☐	☐
Has the issue of confidentiality been explained to you?	☐	☐
Do you understand who will have access to your study information?	☐	☐
Do you understand that we will be video recording the simulation and use for research purposes without revealing identification?	☐	☐

Who explained this study to you?

____

I agree to take part in this study: YES ☐ NO ☐

Signature of Research Subject ____

(Printed Name and Role: Nurse, level of paediatric training)

____

Date:_____________________________

I believe that the person signing this form understands what is involved in the study and voluntarily agrees to participate.

Signature of Investigator _______________________ Date __________

THE INFORMATION SHEET MUST BE ATTACHED TO THIS CONSENT FORM AND A COPY GIVEN TO THE RESEARCH SUBJECT
