# Supplementary material for: Assessment of Visual Attention in Teams with or without Dedicated Team Leaders: A Neonatal Simulation-Based Pilot Randomised Cross-Over Trial Utilising Low-Cost Eye-Tracking Technology
Source: Children (Basel). 2024 Aug 21;11(8):1023. doi: 10.3390/children11081023 (PMC11352304; doi:10.3390/children11081023)
Supplement: Supplementary file 1 [file children-11-01023-s001.zip › children-3143164-Supplementary figure_S1.pdf]

# Study Algorithm

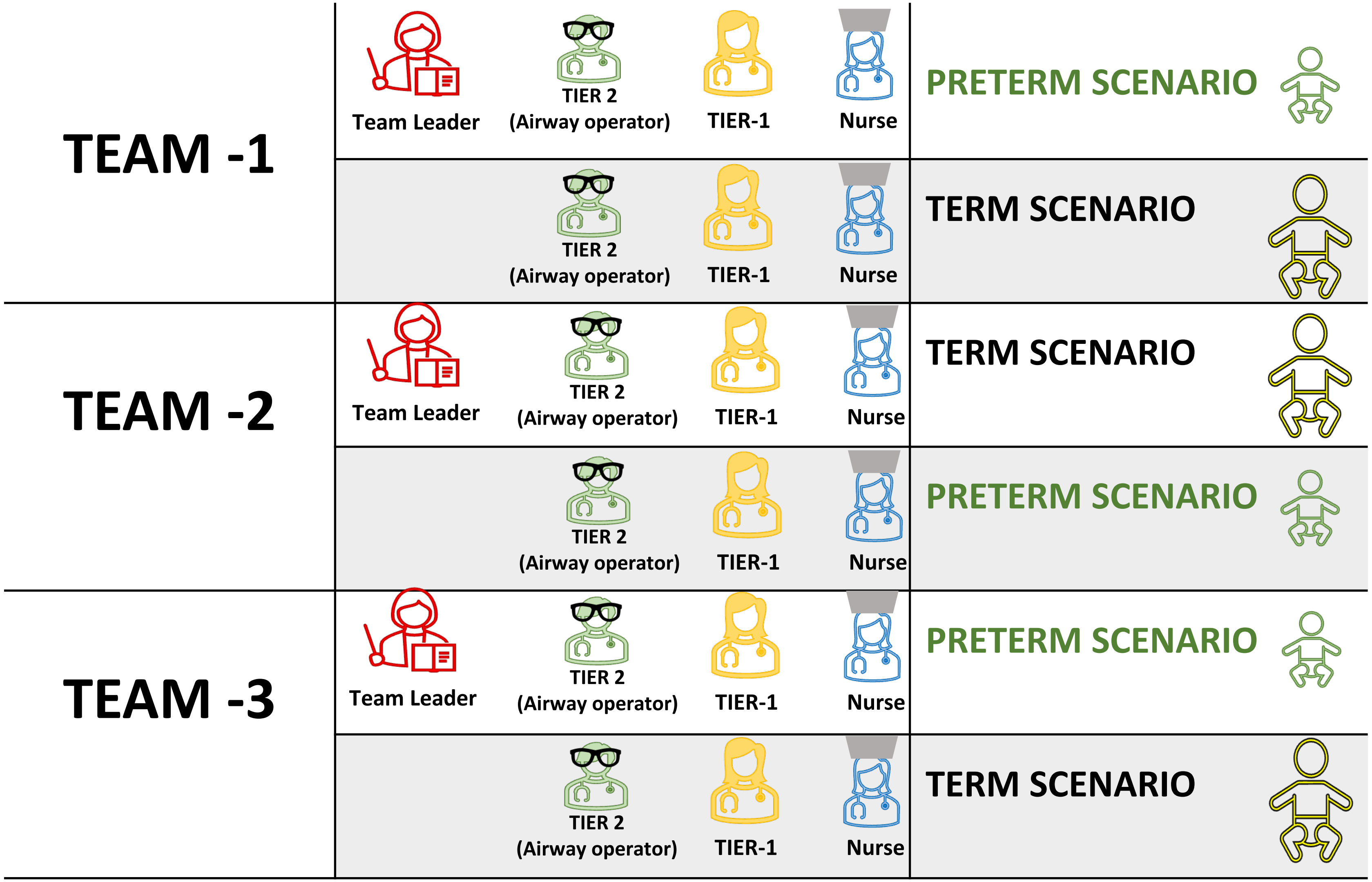


Random selection by tossing a coin for:

- first simulation scenario should be a “Preterm or Term scenario”

- the team should have a team leader or not.

- the selection of who will be the team leader among the two senior trainees.

- The other senior trainee would remain as the airway operator in both simulations.

Following this, the sequence in which each team was allocated with/without a team leader was alternating with each team.
