# Supplementary material for: Assessment of Visual Attention in Teams with or without Dedicated Team Leaders: A Neonatal Simulation-Based Pilot Randomised Cross-Over Trial Utilising Low-Cost Eye-Tracking Technology
Source: Children (Basel). 2024 Aug 21;11(8):1023. doi: 10.3390/children11081023 (PMC11352304; doi:10.3390/children11081023)
Supplement: Supplementary file 1 [file children-11-01023-s001.zip › children-3143164-Supplementary file_S1.pdf]

Study protocol

HUman Factors In Neonatal resuscitation (HUFIN): A simulation based pilot observational study

Prakash Kannan Loganathan[1*], Robert McNicol[2], Matthew Pointon[2], Peter McMeekin[3], Alan Godfrey[2] Micheal Wagner

[1] James Cook University Hospital, South Tees Hospitals NHS Foundation Trust, Middlesbrough, UK
[2] Department of Computer and Information Sciences, Northumbria University, Newcastle upon Tyne, UK
[3] Department of Nursing, Midwifery and Health, Northumbria University, Newcastle upon Tyne, UK

***Corresponding author**
Email: pkannanloganathan@nhs.net

Author contributions
Prakash Kannan Loganathan: Methodology development, manuscript writing and editing
Robert McNicol: Methodology development, manuscript editing
Matthew Pointon: Methodology development, manuscript editing
Peter McMeekin: Methodology development, manuscript editing
Alan Godfrey: Methodology development, manuscript writing and editing

Introduction

Birth asphyxia accounts for a quarter of a million neonatal deaths world-wide but simple resuscitative measures could reduce mortality and morbidities1. Meta-analysis of neonatal resuscitation training (NRT) like the Neonatal resuscitation programme (NRP), Neonatal life support (NLS) and Helping Babies Breathe (HBB) have shown a reduction in perinatal and neonatal mortality when compared with controls. Specifically, NRT decreases the risk of still birth by 21%, 7-day neonatal mortality by 47% and 28-days mortality by 50%[1]. Although there is substantial contribution in reducing mortality by these training programmes, there is a scope to further improvement.

Typically, a resuscitation team commits errors ranging from 23 to 44% during neonatal resuscitation[2]. Errors commonly involve poor airway techniques and not assessing heart rate due to fragmented attention and lack of situational awareness, compromising patient safety during resuscitation[3]. In 2004, a report identified 47 sentinel events contributing to any "any perinatal death or major permanent loss of function unrelated to a congenital condition in an infant with a birth weight >2.5kg". Specifically, the report found the major contributing root causes for sentinel events were poor: (i) staff communication (72%) and (ii) staff competency (47%)[4]. Behavioural skills including lack of teamwork and poor communication as common contributing factors[5]. Accordingly, training processes, staff education and communication protocols are key risk reduction strategies where human factors could be one of the modifiable factors in these situations. Many agencies have emphasised the importance of studying human factors during neonatal resuscitation[6-8]. In spite of the recommendations, there is a dearth of studies in this examining human factors[9].

Multi-tasking and situational awareness

Typically, all individuals within the neonatal medical team (including but not limited to junior trainee doctors, advanced neonatal nurse practitioners and senior trainee doctors) who participate in neonatal resuscitation are NLS programme certified. In practice, it is generally assumed the senior person performs tasks in a more optimal manner and may be (informally) designated with the critically important task of managing the patient's airway, acting as "resuscitation leader". This leads to the designated leader having to multi-task. In dynamic, time critical situations such as neonatal resuscitation, multi-tasking may lead to human error,

loss/reduction in situational awareness, ineffective communication, and sub optimal performance[10]. Alternatively, having a dedicated resuscitation leader who has single dedicated role of managing the team/situation could improve overall situational awareness and resuscitation outcomes. For example, a study analysed resuscitation error and concluded that decreasing cognitive and technical load can be an error reducing strategy[2]. Additionally, a team briefing to empower team members during resuscitation by allocating roles/task could further improve situational awareness and overall performance[10,11]. However, having a stand-alone team leader and use of a team briefing with a standardised checklist is yet to be studied to reduce distractions.

Digital technologies: Cause and solution

Monitoring heart rate, use of pulse oximeter for oxygen saturation, pressure delivered by the resuscitation device (e.g., T piece) and oxygen supply levels are some of the possible multi-tasking requirements during neonatal resuscitation[3]. Previous studies have shown that novice trainees focus more on the monitoring system rather than the patient/baby[3]. Another common issue is task fixation when the user's cognition is impaired by the situation, especially as they are concentrating on a task. Yet, there is no standard or objective methodology for assessing situational awareness but could be inferred by observing human behaviours.

Wearable eye-tracking glasses have been used in aviation industry to study visual attention (VA). Wearable eye-tracking glasses have two cameras, one capturing the reflected infra-red light tracking pupillary movement and another camera capturing video from the participant's viewpoint[12]. This wearable device could provide details on participants visual fixation, gaze shifts (saccades) and regression/lookback (a saccadic movement) whereby the user returns to the task after a distraction. Those eye-tracking data could provide objective and reliable information pertaining to area of interest (AOI), time spent on each AOI and number of gaze shits/saccades during neonatal care. The videos captured by the eye-tracker could also help with the appreciation of the situational context and debriefing the scenario.

Eye-tracking devices/glasses in medicine

Eye-tracking is a method of observing an eye movement as a reflection of attentional behaviour[13]. It is based on the concept that attention is focused to a particular location to

maintain attention on a point of interest. This concept is utilized in eye-tracking technology to capture gaze behaviour, which provides information about an individual's attention over points of interest and allocation over time. In recent years, it has been used in medical field and is a valuable tool in medical education by providing training and effective feedback[13-15].

To date, wearable eye-tracking glasses have been used in few neonatal and critical care studies[3,12,16]. For example, modern wearable eye-tracking glasses have been assessed for usability in a delivery room simulation where focus of cognitive attention was successfully captured, and all wearers agreed they would wear the glasses during the resuscitation[17]. Equally, those findings were replicated in another neonatal simulation study where eye-tracking glasses did not provide any discomfort and participants felt the technology easy to use during the simulation[3]. Attempts to examine human factor have been investigated during neonatal intubation in the neonatal unit, which found 50% of visual attentions is directed away from the infant and the team when they used nonstandard communication[18]. This highlights how eye-tracking device could be an effective tool to analyze human factors for improved medical training, but the study failed to examine task-cognitive load with presence of dedicated team leader and outcomes with use of structured checklist.

We hypothesize that there will be difference in situational awareness, workload, and performance when there is multi-tasking as compared to performing single dedicated role during neonatal care. Here we propose the use of wearable eye-tracking glasses to help standardise approaches to examine situational awareness inferred by objective data from examining human factors/behaviour. Additionally, this may help inform the impact of using standardised checklist and role allocation on multi-tasking, which will be useful in resource limited settings. Subsequently, this manuscript describes the protocol that will be used to evaluate these approaches in a pilot observational study.

Primary aims:

To compare the VA of a team operator/member when s/he:

1) Is managing airway and acting as team leader and
2) Has a dedicated team leader.

Secondary aims:

To examine impact of a team briefing prior to resuscitation on:

1) Team performance using the validated modified neonatal resuscitation performance evaluation (NRPE) tool[19], when (i) the operator is managing both airway and acting as team leader, compared to (ii) having a dedicated team leader with checklist.
2) Compare the workload with or without a team leader and standardised checklist. We will use National Aeronautics and Space Administration task load index (NASA-TLX)[20] for assessing the workload.

Materials and methods

Study

The clinical research question according to the patient, intervention, comparison, outcome, and time (PICOT) process is as follows:

- P: Neonatal resuscitation simulation scenarios, (i) preterm and (ii) term.
- I: Having dedicated airway operator and dedicated "resuscitation leader" (with and without a pre-briefing).
- C: Operator is managing both airway and acting as "resuscitation leader" (with and without a pre-briefing).
- O: Visual attention evaluated by eye-tracking, team performance and workload.
- T: Observational study in a simulation-based model.

Design

We aim to design a single centre, simulation-based, observational study utilising eye-tracking tools for visual attention assessment. The protocol was developed according to the Standard Protocol Items: Recommendations for Interventional Trials' (SPIRIT) checklist[21] as appropriate, Figure 1. As this is a study protocol, no data has been included and conforms to PLOS data policy.

<Figure 1, see end of document>

Study settings

This study will be conducted in regional level-3 neonatal hospital at James Cook University Hospital (South Tees Hospital NHS Foundation Trust), Middlesbrough, UK. Our center has approximately 6000 deliveries with 400 admissions each year. The entire simulation study will be run in a dedicated immersive simulation suite providing standard delivery room equipment and environment.

Recruitment

An ethics application was submitted to IRAS and approved April 2022 (reference number: 314695). Written, informed consent to participate will be obtained by all participants prior to each stage of the study in accordance with General Data Protection Regulations (GDPR). Those interested will then be given a Participant Information Sheet (PIS) concerning the study with consent form (See Appendix-1). All participants will be allowed to withdraw from the study at any time. The individual identity will not be used for any data analysis and reporting.

Participants

Neonatal staff from James Cook Hospital will be invited to participate between April 2022 and August 2022. Participants will not be stratified according to any variable (e.g., age, gender). All nursing and paediatric trainees need to complete Neonatal life support course every 2 years.

Inclusion criteria include:

1. For medical staff: must be paediatric trainee (any level) who has worked at least 6 months in neonatal intensive care settings.
2. For nurse practioners: must be currently working in neonatal unit for at least 6months or more.
3. For nursing staff: must be staff currently working in the neonatal unit.
4. Must have valid NLS certification (completed NLS within the last 3years).
5. Must have participated or observed one neonatal simulation.
6. Must provide written informed consent.

Exclusion criteria include:

1. Not willing to wear eye tracking device.

Team selection: Communications will be via email to recruit interested participants (including nurses, nurse practitioners and doctors). Team selection will occur a few weeks prior to the simulation and will be randomly chosen by placing all written participant names in sealed envelopes and pulled from a ballot box. Teams will have an equal distribution of roles (nurse/tier-1/tier-2) making sure that each consists of:

i. One neonatal nurse,
ii. One tier-1 trainee (senior house officer working as tier-1 with 1-3 years paediatric training or advanced neonatal nurse practitioners working as tier-1) and
iii. Two tier-2 trainees (senior doctors with ≥3years of paediatric training or nurse practitioners working as tier-2).

Outcomes: Primary and secondary

The primary outcomes of this study are the proportion of participants who have altered VA behaviours defined by the objective eye-tracking data (e.g., saccades and fixations). Secondary outcomes are related to the change in team performance and workload due to impact of a team lead with a standardised checklist.

Outcomes: Assessment

Visual attention (situation awareness)

Will be conducted with the wearable eye tracker (Pupil Labs, Core Eye Tracker, Berlin, Germany. 160×51mm, high speed 120Hz and 200Hz, https://pupil-labs.com/products/core). This wearable eye-tracker is modular, easy to clean (disinfect), durable, lightweight, and ergonomically designed for safe use and deployment in any settings. It is fully mobile as it connects to a smartphone for data capture ensuring the wearer is free to move and their normal movement activity are not restricted. Data is wirelessly transferred to Pupil Labs proprietary software and stored locally. Data will then be stored on a secure server where further analysis of those data will be made using a custom-made MATLAB® (MathWorks Inc, Massachusetts, USA) algorithm[22,23]

From all the simulation eye-tracking videos, the following will be analysed:

i. Descriptors pertaining to AOI on: the baby, monitors (e.g., examining pulse oximeter), pressures on the T-piece resuscitation device (*neopuff*), mask technique, oxygen administration, endotracheal intubation (if applicable), performance of procedures like umbilical vein catheterisation, team members, and timer on the resuscitaire.
ii. Time spent on each AOI (indicating VA distribution).
iii. Three eye movements including fixations, saccades and smooth pursuits.

Analysis of eye tracking videos will be performed by the research team. However, one of the limitations of eye tracking is that it does not capture the situations of "Inattentional blindness". Meaning the operator may be looking at some aspect without putting their mind to it. This will be studied by using having an additional camera in the room to gather more (situational) detail in context.

Video

A stationery video camera (camcorder) will record the entire simulation from a fixed position. This will help with the wider and post study assessment by the research team of the primary and secondary objectives. Recording will commence immediately before and cease after the simulation(s). This may help support the assessment of situational impairments beyond the eye tracking video. For understanding the operator's comprehension, interpretation, and interventions we will study the overall team performance and communication.

Team performance

In real time, two investigators who are NLS instructors will independently assess each scenario for team performance. We will use validated modified neonatal resuscitation performance evaluation (NRPE) tool for assessment[19]. This tool was originally developed based on the recommendations of Neonatal resuscitation programme, American Heart Association. NRPE provides categorical score (yes=1 and no/incomplete action=0) for each of the actions. It marks the performance under three domains: (i) appropriateness of decision making, (ii) technical skills and (iii) time taken to perform the appropriate resuscitative measures. It has seven sub domains: (i) preparation and initial steps, (ii) communication of heart rate, (iii) bag/mask ventilation, (iv) chest compressions, (v) intubation, (vi) medication administration and (vii) umbilical vessel catheterisation. Results from this tool have been shown to be valid and with high inter-rater reliability[24].

We have modified NRPE tool to match recommendations of Neonatal life support in the UK. Modifications would include use of two sets of inflation breaths, use of ventilation breaths and other minor modifications (Appendix 2). NLS, UK recommends two sets of 5 inflation breaths as compared to timed action as per NRP (30 seconds of Positive pressure ventilation) and recommends logical sequence of action followed by assessment. For these reasons, we have removed the domain for "time taken to perform the appropriate resuscitative measures". This tool's content validity is assured as the tool originally by expert neonatologist and modified by practising neonatologist (PL). Response process validity controlling for potential sources of error with administration will be minimised by using standardised simulation environment, using the same two simulation scenarios, and maintaining the similar team structure for all the simulations. We would calculate inter-rater reliability by two independent NLS instructors which would provide internal validity.

Workload

For assessment of workload we will use the National Aeronautics and Space Administration task load index (NASA-TLX)[20]. This scoring system includes 6 scales on how an individual experiences work demands in various dimensions: Mental Demand (MD), Physical Demand (PD) and Temporal Demand (TD), Frustration (FR), Effort (EF), and Performance (PE) (Appendix 3).This self-assessment scoring system has been used more than 300 studies and used in various industries like aviation, defence and recently in health care[25]. Reliability and validity of NASA-TLX has been demonstrated in studies[26]. We will use raw scores of NASA-TLX rather than weighted scores as raw score is more time efficient and simpler to apply[27]. After completing each simulation, participants would be asked to complete NASA-TLX forms. In this form, there would be definitions for each rating scale and for each domain. For each of the six domains the participants would mark their feeling in the scale of 1 to 20, in each domain. 20 being the worst experience and 1 being the best experience

Outcomes: Data

Participants

Participant gender and age will be recorded. Additionally, data pertaining to participant professional training and neonatal experience will also be gathered prior to the simulation, Table S1.

Table S1: Participant data

	Nurses	Team leader	Doctors
Training / Nurse band (level)			
Neonatal experience (years)			
Times completed NLS course (number)			
Completed NLS within last 1 year (yes/no)			
Prior neonatal simulation experiences (number)			
Prior neonatal resuscitation real life experiences (number)			

Visual attention

During each simulation participant visual attention will be objectively assessed with a wearable eye-tracker, as previously described.

Table S2: Visual attention outcomes

	All scenarios (Between participant comparison)			Within team post comparison (Same person comparison)		
	Nurse	Tier-1	Tier-2	Nurse	Tier-1	Tier-2
AOI (description)						
Time on AOI (seconds)						
Time-to-first fixation (seconds)						
Saccades (mean and variability)						
Fixations (mean and variability)						
Smooth pursuits (mean and variability)						

Simulation

Prior to each simulation, participants will receive briefing about features of simulation dolls (i.e., to mimic the patient), how they need to assess the doll, and what information they need to obtain from the simulation instructors. Before each scenario each team will have approximately five minutes to prepare for the simulation. We will have two scenarios, one with management of an extreme preterm infant and another with the management of a term newborn with complications at delivery. Details of term and preterm resuscitation scenarios are provided in Appendix-1 and Appendix-2. Both these scenarios were used previously in our neonatal unit simulations. Both the simulation scenarios will need endotracheal intubation with the term baby scenario needing chest compression and umbilical vein administration of epinephrine. We will use the SimNewB (Laerdal Medical, Laerdal, Stavanger, Norway) low

fidelity manikin for term and Premature infant Anne-low fidelity (Laerdal Medical, Laerdal, Stavanger, Norway) for the preterm scenarios, respectively. The two scenarios will be the same for all participants. We will run the two simulation scenarios with three different teams, giving a total of 6 scenarios. Each scenario will be running approximately for 10-15 minutes.

Study algorithm

Each team will participate in two simulation scenarios (i) term and (ii) preterm. With 3 teams, there will be total of 6 simulation experiments. Each team will undergo one scenario with a team leader and another scenario without a team leader. The sequence in which each team is allocated with/without team leader would be alternating with each team. For example, if team #1 starts with team leader for their preterm scenario, team #2 would participate same preterm scenario without team leader. This will ensure that both scenarios have an equal number of scenarios with and without a team leader.

Briefing

All scenarios will be preceded by locally developed standardised briefing including checklist of role allocation, equipment checking, and escalation plan. Most neonatal units will have this kind of checklist with some modifications to suit local set up. All the airway operators will be briefed about using eye-tracking wearable and undergo pre-simulation test for familiarity. Briefing will be done by the team leader, and we will use a local unit briefing checklist (Appendix-3).

Debriefing

After completing the scenario, team will be provided debrief immediately after completing the simulation. All the debrief would be provided by the same investigator (NLS instructor) who is not involved in the outcome assessment. We would use local unit debrief checklist developed based on the method of debrief as described by Skare et al[19]. This method has three phases: descriptive, analytical, and reflective (Appendix-4). During debrief, we will collect feedback with regards: 1) How did the simulation went? 2) What were the good things? 3) Things to improve 4) How they felt during the simulation?

Sample size

In a previous study using eye tracking glasses examining VA during neonatal resuscitation, in which the operator acted both as an airway manager and team leader[12]. In this study operator spent 35% (SD 8) of time focused on the infant. With the presence of dedicated resuscitation leader, to improve the VA on the infant to 55% with 80% power and alpha error of 0.05, we will need to conduct 6 scenarios with 3 samples in each group (equalling 18 participants).

Statistics

This is an exploratory study consisting of XXX groups. To the authors knowledge, there has yet to be a comprehensive analysis of VA with eye-tracking in neonatal simulations examining impact of a team lead with a checklist. However, there has been a pilot study using eye-tracking in neonatal resuscitation which used a dataset of 6 participant video-based eye-tracking recordings of 5mins duration[12]. Therefore, our anticipated dataset size of 24 participants, will provide greater insight to examine VA behaviour differences and detect impact of a tam lead with a standardised checklist. Data will be analysed in SPSS (v23, IBM) and R studio (R. RStudio, Boston, MA, USA). To conform to the exploratory nature of the work, descriptive statistics such as means, and ranges will be used here based on the number of participants stratified across 4 teams. Independent t-tests will further explore any observable differences. Those data will be complimented with simple plots. Anonymised data will be made available on reasonable request.

Discussion

Here we provide a protocol for eye-tracking in neonatal resuscitation, with a focus on examining the impact of a team lead with a standardised checklist. At present there is no gold standard or proposed method for incorporating a team lead and/or checklist into neonatal resuscitation. This protocol will allow consideration of the combined and interactive impact of a team lead with a checklist for the field, considering the attentional demands using wearable eye-tracking video data to collect objective data to examine behaviour through visual attention.

This assessment paradigm distinguishes therefore itself from other work in the field. To the authors knowledge, no research has examined impact of a team lead with a checklist through use of a wearable eye-tracker, while also considering the scenarios of term and preterm in neonatal resuscitation. Furthermore, there hasn't been attempts explore team performance and workload. Therefore, the development and synthesis of this protocol will provide an important step in quantitively assessing behaviour for appropriate clinical practice in neonatal resuscitation.

This protocol does carry some limitations. Firstly, there are several equivalent technologies that could be used for objective eye-tracking. However, given the lack of protocols in neonatal resuscitation, we feel the proposed manuscript provides a pragmatic starting point to work on and develop in future research.

Conclusions

References

1. Patel A, Khatib MN, Kurhe K, Bhargava S, Bang A. Impact of neonatal resuscitation trainings on neonatal and perinatal mortality: a systematic review and meta-analysis. *BMJ Paediatr Open.* 2017;1(1):e000183.
2. Yamada NK, Yaeger KA, Halamek LP. Analysis and classification of errors made by teams during neonatal resuscitation. *Resuscitation.* 2015;96:109-113.
3. Wagner M, Gröpel P, Bibl K, Olischar M, Auerbach MA, Gross IT. Eye-tracking during simulation-based neonatal airway management. *Pediatr Res.* 2020;87(3):518-522.
4. Sentinel event alert issue 30--July 21, 2004. Preventing infant death and injury during delivery. *Adv Neonatal Care.* 2004;4(4):180-181.
5. Thomas EJ, Sexton JB, Helmreich RL. Translating teamwork behaviours from aviation to healthcare: development of behavioural markers for neonatal resuscitation. *Qual Saf Health Care.* 2004;13 Suppl 1(Suppl 1):i57-64.
6. (UK) RC. NLS-Newborn Life Support. 2021.
7. Aziz K, Lee HC, Escobedo MB, et al. Part 5: Neonatal Resuscitation: 2020 American Heart Association Guidelines for Cardiopulmonary Resuscitation and Emergency Cardiovascular Care. *Circulation.* 2020;142(16_suppl_2):S524-s550.
8. Madar J, Roehr CC, Ainsworth S, et al. European Resuscitation Council Guidelines 2021: Newborn resuscitation and support of transition of infants at birth. *Resuscitation.* 2021;161:291-326.
9. Pediatrics AAo. https://services.aap.org/en/learning/neonatal-resuscitation-program/nrp-research-grant-and-young-investigator-award-program/. Accessed 23rd March, 2021.
10. Brennan PA, Holden C, Shaw G, Morris S, Oeppen RS. Leading article: What can we do to improve individual and team situational awareness to benefit patient safety? *Br J Oral Maxillofac Surg.* 2020;58(4):404-408.
11. Litke-Wager C, Delaney H, Mu T, Sawyer T. Impact of Task-Oriented Role Assignment on Neonatal Resuscitation Performance: A Simulation-Based Randomized Controlled Trial. *Am J Perinatol.* 2021;38(9):914-921.
12. Law BHY, Cheung PY, Wagner M, van Os S, Zheng B, Schmölzer G. Analysis of neonatal resuscitation using eye tracking: a pilot study. *Arch Dis Child Fetal Neonatal Ed.* 2018;103(1):F82-f84.
13. Ashraf H, Sodergren MH, Merali N, Mylonas G, Singh H, Darzi A. Eye-tracking technology in medical education: A systematic review. *Med Teach.* 2018;40(1):62-69.
14. Merali N, Veeramootoo D, Singh S. Eye-Tracking Technology in Surgical Training. *J Invest Surg.* 2019;32(7):587-593.
15. Stuart S, Alcock L, Godfrey A, Lord S, Rochester L, Galna B. Accuracy and re-test reliability of mobile eye-tracking in Parkinson's disease and older adults. *Medical Engineering & Physics.* 2016;38(3):308-315.
16. Henneman EA, Cunningham H, Fisher DL, et al. Eye tracking as a debriefing mechanism in the simulated setting improves patient safety practices. *Dimens Crit Care Nurs.* 2014;33(3):129-135.
17. Katz TA, Weinberg DD, Fishman CE, et al. Visual attention on a respiratory function monitor during simulated neonatal resuscitation: an eye-tracking study. *Arch Dis Child Fetal Neonatal Ed.* 2019;104(3):F259-f264.

18. Law BHY, Schmölzer GM. Analysis of visual attention and team communications during neonatal endotracheal intubations using eye-tracking: An observational study. *Resuscitation.* 2020;153:176-182.
19. Skåre C, Calisch TE, Saeter E, et al. Implementation and effectiveness of a video-based debriefing programme for neonatal resuscitation. *Acta Anaesthesiol Scand.* 2018;62(3):394-403.
20. Hart S. Development of NASA-TLX: Results of empirical and theoretical research." inP. A. Hancock and N. Meshkati (eds.), Human Mental Workload. In: North-Holland; 1988.
21. Chan A-W, Tetzlaff JM, Gøtzsche PC, et al. SPIRIT 2013 explanation and elaboration: guidance for protocols of clinical trials. *BMJ : British Medical Journal.* 2013;346:e7586.
22. Anzalone AJ, Blueitt D, Case T, et al. A Positive Vestibular/Ocular Motor Screening (VOMS) Is Associated With Increased Recovery Time After Sports-Related Concussion in Youth and Adolescent Athletes. *The American Journal of Sports Medicine.* 2017;45(2):474-479.
23. Stuart S, Hickey A, Vitorio R, et al. Eye-tracker algorithms to detect saccades during static and dynamic tasks: a structured review. *Physiological Measurement.* 2019;40(2):02TR01.
24. Sawyer T, Leonard D, Sierocka-Castaneda A, Chan D, Thompson M. Correlations between technical skills and behavioral skills in simulated neonatal resuscitations. *Journal of Perinatology.* 2014;34(10):781-786.
25. Hart SG. NASA-task load index (NASA-TLX); 20 years later. Paper presented at: Proceedings of the human factors and ergonomics society annual meeting2006.
26. Hoonakker P, Carayon P, Gurses A, et al. MEASURING WORKLOAD OF ICU NURSES WITH A QUESTIONNAIRE SURVEY: THE NASA TASK LOAD INDEX (TLX). *IIE Trans Healthc Syst Eng.* 2011;1(2):131-143.
27. Said S, Gozdzik M, Roche TR, et al. Validation of the Raw National Aeronautics and Space Administration Task Load Index (NASA-TLX) Questionnaire to Assess Perceived Workload in Patient Monitoring Tasks: Pooled Analysis Study Using Mixed Models. *Journal of medical Internet research.* 2020;22(9):e19472.

Figure S1. SPIRIT Figure

	**STUDY PERIOD**						
	**Enrolment**	**Allocation**	**Post-allocation**				**Close-out**
**TIMEPOINT****	**-t_1**	**0**	***t_1* *(with team lead)***	***t_2* *(no team lead)***	***t_3)***	***t_4***	***t_5***
**ENROLMENT:**							
**Eligibility screen**	X						
**Informed consent**	X						
**Allocation**		X					
**INTERVENTIONS: NA**							
**ASSESSMENTS:**							
Participant details		X					
Visual attention			X	X			
Team performance			X	X			
Workload			X	X			

*Recommended content can be displayed using various schematic formats. See SPIRIT 2013 Explanation and Elaboration for examples from protocols.
**List specific timepoints in this row.
