# Supplementary material for: Assessment of Visual Attention in Teams with or without Dedicated Team Leaders: A Neonatal Simulation-Based Pilot Randomised Cross-Over Trial Utilising Low-Cost Eye-Tracking Technology
Source: Children (Basel). 2024 Aug 21;11(8):1023. doi: 10.3390/children11081023 (PMC11352304; doi:10.3390/children11081023)
Supplement: Supplementary file 1 [file children-11-01023-s001.zip › children-3143164-Supplementary tables-S1_S2_S3.pdf]

Supplemental table S1: NRPE and BAT scores for each scenarios by two assessors						
**NRPE scores**						
Team Leader						
Assessor-1				Assessor-2		
	Decision	Technique	Total	Decision	Technique	Total
Preterm	100	100	100	80	100	95
Preterm	100	93	95	80	100	95
Term	100	93	95	85.7	94	92
No Team Leader						
Assessor-1				Assessor-2		
	Decision	Technique	Total	Decision	Technique	Total
Preterm	100	94	95	100	93	95
Term	100	93	95	100	89	92
Term	100	93	95	100	89	92
**BAT scores**						
Team Leader						
	Assessor-1			Assessor-2		
	Total (Max 44)	Percentage		Total	Percentage	
Preterm	39	87		29	66	
Preterm	38	86		31	70	
Term	44	100		44	100	
No Team Leader						
	Assessor-1			Assessor-2		
	Total	Percentage		Total	Percentage	
Preterm	37	84		40	91	
Term	43	98		30	68	
Term	39	89		34	77	

Supplemental table S2: NASA-TASK LOAD INDEX-Self reporting in participants who were airway operators and wearing eye-tracking glasses						
	Participant 1		Participant 2		Participant 3	
	No Team Leader	Team leader	No Team Leader	Team leader	No Team Leader	Team leader
Mental Demand	17	9	17	5	15	11
Physical demand	12	5	5	5	15	3
Temporal demand	16	9	5	5	15	10
Performance	7	5	5	5	10	4
Effort	12	9	5	5	16	5

Frustration level	17	4	4	5	17	2

**Supplemental table S3: Individual NASA-TASK LOAD INDEX scores (after excluding airway operators)**						
	Mental demand	Physical demand	Temporal demand	Performance	Effort	Frustration level
Team Leader						
Preterm						
Nurse	10	2	4	14	10	15
Nurse	10	10	16	5	11	3
Junior trainee	20	2	16	10	16	5
Junior trainee	5	2	3	6	4	1
Junior trainee	14	5	5	1	3	2
Senior trainee	17	5	15	4	15	1
Term						
Nurse	16	9	16	13	13	16
Junior trainee	14	9	8	2	16	1
Senior trainee	18	9	14	5	15	10
No Team Leader						
Preterm						
Nurse	14	3	14	9	15	9
Junior trainee	16	4	3	2	2	4
Term						
Nurse	18	10	16	14	13	12
Nurse	15	16	16	5	16	5
Junior trainee	13	9	5	3	5	3
Senior trainee	9	11	9	16	14	1
