# Supplementary material for: Assessment of Visual Attention in Teams with or without Dedicated Team Leaders: A Neonatal Simulation-Based Pilot Randomised Cross-Over Trial Utilising Low-Cost Eye-Tracking Technology
Source: Children (Basel). 2024 Aug 21;11(8):1023. doi: 10.3390/children11081023 (PMC11352304; doi:10.3390/children11081023)
Supplement: Supplementary file 1 [file children-11-01023-s001.zip › children-3143164-tex.zip!/Definitions/logo-mdpi-eps-converted-to.pdf]

MDPI
